# Differences in life expectancy between olympic high jumpers, discus throwers, marathon and 100 meter runners

**DOI:** 10.1186/s13102-017-0067-z

**Published:** 2017-01-26

**Authors:** Jeffrey Lee-Heidenreich, David Lee-Heidenreich, Jonathan Myers

**Affiliations:** 1 Henry M. Gunn High School, Palo Alto, CA USA; 2 School of Arts and Sciences, Stanford University, Stanford, CA USA; 3 VA Palo Alto Health Care System, Palo Alto, CA USA; 4 Department of Medicine, Stanford University, Stanford, CA USA; 5 Mail Code 111C, 3801 Miranda Avenue, Palo Alto, CA USA 94304; 6 797 Mayview Avenue, Palo Alto, CA USA 94303

**Keywords:** Survival, Body weight, Athletes

## Abstract

**Background:**

Several studies have demonstrated that body habitus is associated with survival (life expectancy) time. We sought to determine if survival differed between elite athletes with a range of body types. We hypothesized that the survival would differ between athlete types and that ectomorph athletes would have longer survival than heavier athletes.

**Methods:**

For each Olympics between 1928 and 1948 we identified the top (up to 20) Olympic male and female finishers in the high jump (HJ), discus throw, marathon, and 100-m run. We determined date of death using internet searches and calculated age-specific expected survival using published US life tables. We adjusted life expectancy for country of origin based on Global Burden of Disease data.

**Results:**

We identified a death date for 336 of 429 (78%) Olympic athletes including 229 males (55 marathon, 56 100-m 58 high jump, 60 discus), and 107 females (54 100-m, 25 high jump, 28 discus). Discus throwers were heaviest and marathon runners the lightest and oldest athletes (*p* < 0.01). Observed-expected survival was highest for high jumpers (7.1 years for women, 3.7 years for men) and marathon runners (4.7 years for men) and lowest for sprinters (−1.6 years for women and −0.9 years for men). In multivariate analysis controlling for age and gender, type of sport remained significantly associated with mortality with greatest survival for high jumpers and marathon runners compared to discus throwers and sprinters (*p* = 0.005). Controlling for weight, reduced the survival benefit of high jumpers over discus throwers, but had little effect on the survival benefit of marathon runners vs. sprinters.

**Conclusion:**

Significant differences in long term survival exist for different types of track and field Olympic athletes that were explained in part by weight.

## Background

Studies of the mortality of elite athletes compared to the general population have shown favorable outcome for many, but not all, sports. French Olympic rowers showed increased longevity compared to the general French population matched on age and birth year [1]. In contrast, studies involving New Zealand rugby players [2] and Major League Baseball players [3] have shown similar mortality with the general population. A study of German international soccer players demonstrated shorter life expectancy compared to age-matched Germans [4]. The reasons for the different outcomes are not clear, but may relate to body habitus [5].

Several studies have demonstrated that body habitus is associated with survival, with higher body mass index (BMI) being associated with increased mortality in the general population [6]. Elite power lifters (known to have a high BMI) have been shown to have a higher mortality than the general population in several studies [7, 8].

We sought to determine if survival differed between elite (Olympic) ectomorph (high jump, marathon) and mesomorph (discus, 100 m run) athletes. We hypothesized that the ectomorph athletes would have longer survival but that the mortality difference would be largely explained by body habitus.

## Methods

### Subjects

We used publically available data to identify the top 20 males and females in the Olympic high jump, discus, marathon and 100 m run from 1928–1948 [9]. Marathon (and other long-distance races) were only male events during this time period. Through these data, we identified age at competition, height, weight, country of origin, and date of death. If an athlete was in more than one Olympic Games we used their first Olympic participation.

### Outcome measures

- *All-cause mortality*: Death dates were determined from publically available websites, including country specific Olympic websites or personal pages/Wikipedia.
- *Expected survival*: We obtained age, gender and decade of birth-specific survival from US census data.
- *Adjustment for country of origin*: We determined country-specific relative rates of mortality using Global Burden of Disease Data using the US as the reference (relative rate of 1.0) [10]. An athlete’s life expectancy was calculated by multiplying the US age, gender and decade of birth specific mortality by the relative rate of survival of the athlete’s country of origin.

### Statistical analysis

Baseline characteristics were compared between groups using the Pearson chi-square test for categorical variables and the t-test for continuous variables. Categorical variables were reported as percentages and continuous variables were reported as mean, standard deviation, median, and interquartile range. A *p*-value of <0.05 was considered significant for all tests. We used proportional hazards analysis to determine the impact of athlete type on survival adjusting for age, height, weight, and gender. Year of Olympics, and receiving a medal (yes/no) were not found to be significant in univariate analyses and were not examined in additional models. Interactions were tested between sport event, gender and mortality. The authors are solely responsible for the design and conduct of this study, including all analyses, drafting and editing, and its final contents. All analyses were performed using R software.

## Results

We identified a death date for 336 of 429 (78%) Olympic athletes including 229 males (55 marathon, 56 100-m 58 high jump, 60 discus), and 107 females (54 100-m, 25 high jump, 28 discus). Marathon runners (men) followed by discus throwers were oldest at the time of Olympics (Tables 1, 2). Discus throwers were heavier than other athletes. High jumpers and discus throwers were more likely to be from the United States than sprinters (women) or marathon runners (men) though the differences did not reach statistical significance.

**Table 1:** Characteristics of olympic male athletes by event

	Marathon	100-m	High Jump	Discus	*P* value
All (N)	55	56	58	56	
Age at race (years)	29.7 ± 5.9	23.8 ± 3.0	22.7 ± 3.1	26.0 ± 4.5	<0.001
Weight (lbs)	136.8 (62.1 kg) ± 12.3 (5.4 kg)	156.6 (71.1 kg) ± 12.1 (5.5 kg)	165.0 (74.9 kg) ± 17.7 (8.0 kg)	207.9 (94.4 kg) ± 20.2 (9.2 kg)	<0.001
Height (in)	67.0 (1.70 m) ± 1.6 (0.041 m)	69.7 (1.77 m) ± 2.0 (0.051 m)	72.1 (1.83 m) ± 3.1 (0.079 m)	73.5 (1.87 m) ± 2.2 (0.056 m)	<0.001
USA athlete (%)	5.5	17.9	19.0	20.0	0.12

**Table 2:** Characteristics of female olympic athletes by event

	100-m	High Jump	Discus	*P* value
All (N)	54	25	28	
Age at race (years)	20.7 ± 3.1	20.1 ± 3.4	22.2 ± 3.3	0.041
Weight (lbs)	127 (57.7 kg) ± 17.8 (8.1 kg)	131 (59.5 kg) ± 9.3 (4.2 kg)	149 (67.6 kg) ± 16.6 (7.5 kg)	<0.001
Height (in)	65.6 (1.67 m) ± 2.3 (0.058 m)	67.1 (1.70 m) ± 2.1 (0.053 m)	66.9 (1.70 m) ± 2.3 (0.058 m)	0.056
USA athlete (%)	13.0	24.0	21.4	0.41

### Survival

#### Unadjusted survival

**Fig. 1:**
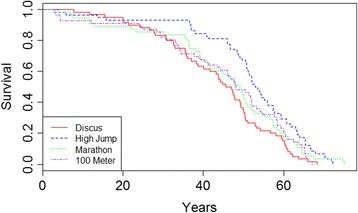
Survival following the Olympics is shown for male athletes by event

**Fig. 2:**
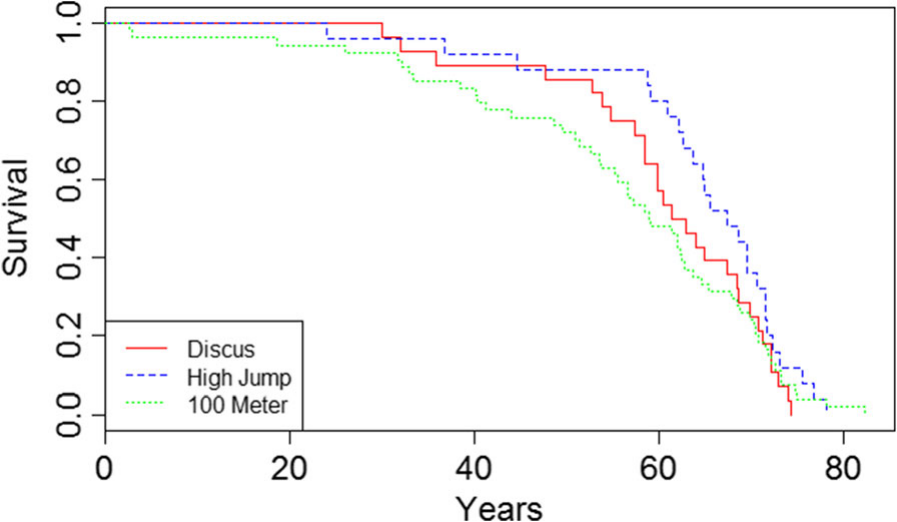
Survival following the Olympics is shown for female athletes by event

Unadjusted survival was higher for high jumpers compared to discus throwers (Fig. 1). The survival difference was noted for both men (Fig. 2a) and women (Fig. 2b).

#### Adjusted survival

We adjusted for athlete characteristics using two methods (observed – expected life expectancy of the population using age, sex, year of birth and country of origin) and proportional hazards. Observed-expected survival was greatest for high jumpers (7.1 years for women, 3.7 years for men) and marathon runners (4.7 years for men) and lowest for sprinters (−1.6 years for women and −0.9 years for men, Table 3).

**Table 3:** Survival for olympic athletes by event

	Marathon	100-m	High Jump	Discus	*P* value
Males					
Obs. Survival	45.4 ± 17.3	45.2 ± 17.3	51.7 ± 14.8	43.6 ± 14.2	0.03
Expected	40.7 ± 5.8	46.1 ± 4.9	48.0 ± 4.1	44.3 ± 4.7	<0.0001
Obs-Exp	4.7 ± 16.1	–0.9 ± 18.3	3.7 ± 14.9	–0.6 ± 13.9	0.13
Females					
Obs. Survival	N/A	55.6 ± 17.8	64.2 ± 12.5	60.6 ± 12.1	0.06
Expected	N/A	57.2 ± 3.4	57.1 ± 3.9	53.9 ± 4.2	<0.0001
Obs-Exp	N/A	–1.6 ± 17	7.1 ± 13.4	6.9 ± 12.3	0.01

Observed (obs) -Expected (exp) is the average difference for individuals and many not equal the difference in averages of observed and expected due in part to rounding

In multivariable proportional hazard analysis, age (hazard ratio 1.08, 95% CI: 1.05–1.11 per year of age) and gender (HR 2.07, 95% CI: 1.58–2.71), but not year of Olympics was significantly associated with survival. When the sport event was added to the model of age and gender all three remained significant (sport event *p* = 0.005, age *p* < 0.0001, gender *p* < 0.001). Using 100°m sprinters as the reference, the adjusted hazard ratio for mortality was similar for discus throwers and lower for high jumpers and marathon runners (Table 4). There was no significant interaction between gender, sporting event and adjusted hazard ratio for mortality.

**Table 4:** Impact of adjusting for weight on survival differences by event

	Hazard Ratio for Mortality (95% Confidence Interval)			
Model	Marathon	100-m	High Jump	Discus
Adjusted for Age at event, Gender,	0.60 (0.51–0.87)	Reference	0.71 (0.53–0.95)	1.00 (0.75–1.34)
Adjusted for Age at event, Gender and Weight	0.67 (0.45–1.02)	Reference	0.70 (0.52–0.93)	0.83 (0.55–1.25)

N/A not applicable, the marathon was not an Olympic evesnt for females during the study period

#### Weight and survival

Weight was independently associated with mortality after adjustment for age and gender (HR for mortality per 10 lb increase: 1.06, 95% CI 1.02–1.11). The results were similar with and without imputation. With weight added to the multivariable proportional hazards model along with age and gender, the survival benefit for high jumpers compared to 100 m sprinters was unchanged (Table 4) but was attenuated for high jumpers vs. discus throwers (HR 0.71, 95% CI: 0.52–0.97, adjusted for age and gender vs. HR 0.84 95% CI: 0.56–1.27 with adjustment for age, gender and weight). However, an interaction term of weight*sport was not significantly associated with mortality (adjusted for age and gender). While increased height was associated with mortality after adjustment for age and gender (HR for a one inch increase in height: 1.04, 95% 1.01–1.08), height was correlated with weight and after adjustment for weight, height was no longer significantly associated with mortality.

#### Olympic medal and survival

Medal winners had similar risk of mortality compared to non-medal winners after controlling for age, gender and event (HR 1.12, 95% CI: 0.84–1.50).

## Discussion

Our study shows that life-expectancy for Olympians varies by event with high-jumpers and marathon runners living longer than discus throwers and sprinters. There were also large differences in weight between athlete types which explained some of the differences in survival, particularly between high-jumpers and discus throwers. High jumpers were lighter than discus throwers and lighter athletes, in general, lived longer than heavier athletes. In addition, high jumpers and marathon runners lived longer than similarly aged members of the general population for both men women. Female, but not male, discus throwers lived longer than the general population. There was no survival benefit for sprinters compared to the general population.

Past research has evaluated the survival of Olympic athletes in comparison with the general public. A systematic review by Lemez found that survival for athletes was better than the general population in many but not all studies (5). In a recent study by Antero-Jacquemin et al. [1], Olympic rowers lived longer than the general French population. The standardized mortality ratio (SMR) was 0.58 (95% confidence interval: 0.43–0.78) indicating that mortality was 42% lower for the Olympic athletes than the average French resident. Deaths from cardiovascular disease were also lower than expected for Olympic athletes in this study (SMR 0.41 (95% CI 0.16–0.84)). There was a trend toward reduced cancer mortality (SMR 0.59 (95% CI 0.29–1.07)). Other causes of death were no different between Olympic athletes and the general population of France. Our study was consistent with these findings, but shows that certain athlete types (high jumpers and marathon runners) but not others (sprinters) live longer than the general populations of their respective countries.

Among Olympic athletes, those winning medals may have the best outcome. In a study by Clarke, Olympic medalists lived longer than the general population regardless of country. [11] Specifically, Olympic medalists lived an average of 2.8 years longer than controls of the general population. There was no survival advantage by type of medal (Gold, Silver, and Bronze) though the study had limited power to detect differences in these groups. Those who medaled in endurance sports (long distance running, cross-country skiing) and mixed sports (track and field jumping, soccer, ice hockey, basketball, and short distance running) had a greater survival advantage than medalists in power sports (field throwing, weightlifting, wrestling, and boxing). We did not observe a difference in outcome between medalists and non-medalists though the sample size limited our ability to find small to moderate differences.

One of the main differences between the athlete types was weight, and to a lesser extent height. This difference in weight is important in interpreting survival differences between athlete types because weight was associated with mortality in our study as well as in others [12–14] The impact on mortality may depend on athlete age with lighter weight being more beneficial for the young than for the elderly. Dahl et al. [12] reported that individuals between 70–95 years with a higher BMI had approximately 20% lower mortality than those with lower BMI. A similar study by Flicker et al. [13] showed that older individuals with higher BMI had lower mortality related to heart disease. However, Rosengren et al. [14] reported that among young to middle aged individuals, weight gain after the age of 20 raised a person’s risk of coronary death. Our study is consistent with the latter, in which lighter athletes had greater survival than heavier athletes. This difference in weight explained much of the mortality difference between high jumpers and discus throwers though it explained little of the survival difference between marathon runners and sprinters.

Numerous public health organizations have established recommendations for ideal body weight. For example, the World Health Organization recommended that individuals maintain a body mass index of 18 – 25 kg per meters squared [15]. This recommendation is independent of age, based on findings that in midlife, a higher BMI is associated with higher mortality. However, an “obesity paradox” exists for particularly elderly groups, in which higher weight is associated with better survival. Ideal body weight may also differ for elite athletes. For example, heavier elite athletes may have an extremely low percentage of body fat, and thus the relationship between weight and survival may be attenuated. While we do not know the mechanism for increased mortality for different athlete types there are several implications for athletes and coaches. Those athletes with a larger body habitus may have more weight gain following competitive athletics and associated complications such as metabolic syndrome and diabetes. Larger athletes can be counseled that maintaining a healthy lifestyle, while recommended for all, may be of particular importance for them.

### Limitations

Our study has several potential limitations. We were unable to control for socioeconomic status within countries which is known to be associated with survival [16, 17]. It is possible that discus throwers had a lower socio-economic status than high-jumpers, and this may have explained some of the difference in mortality. Our power was limited to detect interactions between athlete characteristics (e.g. gender and sport) and small to moderate interactions may exist. In addition, cause of death was not available, and there may be differences in cardiovascular or cancer mortality between the two athlete types. There may be benefits related to being an Olympian that are unrelated to training and health behaviors and additional investigations are needed to explore these potential mechanisms [18]. Changes in health behaviors and medical care over time may impact health status and future studies are needed to determine how recent trends are impacting mortality. Finally, women were not eligible for long-distance races during the Olympics we examined.

## Conclusion

In conclusion, our study demonstrated differences in survival by elite athlete type. Olympic high jumpers and marathon runners live longer than elite sprinters. This difference was explained in part by differences in body habitus as heavier athletes had worse outcomes than lighter athletes. Controlling for weight, reduced the survival benefit of high jumpers over discus throwers, but had little effect on the survival benefit of marathon runners vs. sprinters. Future studies are needed to determine if there are differences in cause-specific mortality (e.g. cardiovascular disease) between different types of elite athletes.

## Keypoints

- Olympic high jumpers and marathon runners lived longer than the general population of similar age, gender and birth year.
- 100 m runners did not live longer than the general population.
- Difference in survival between high jumpers and discus throwers are explained by lower weight of high jumpers

## Abbreviations

BMI: Body mass index
CI: Confidence interval
HJ: High jump
HR: Hazards ratio
SMR: Standardized mortality ratio
